# Muscle B mode ultrasound and shear-wave elastography in idiopathic inflammatory myopathies (SWIM): criterion validation against MRI and muscle biopsy findings in an incident patient cohort

**DOI:** 10.1186/s41927-022-00276-w

**Published:** 2022-08-08

**Authors:** Shereen Paramalingam, Merrilee Needham, Sarah Harris, Susan O’Hanlon, Frank Mastaglia, Helen Keen

**Affiliations:** 1 University of Notre Dame Australia, Fremantle, WA Australia; 2 Department of Rheumatology, Fiona Stanley Hospital, 11 Robin Warren Dr, Murdoch, WA 6150 Australia; 3 Institute for Immunology and Infectious Diseases, Murdoch University, Murdoch, WA Australia; 4 Department of Neurology, Fiona Stanley Hospital, Murdoch, WA Australia; 5 Institute for Health Research, Notre Dame Australia, Fremantle, WA Australia; 6 Western Australian Health Translation Network (WAHTN), Bentley, WA Australia; 7 Department of Radiology, Fiona Stanley Hospital, Murdoch, WA Australia; 8 Present Address: Perron Institute for Neurological and Translational Science, University of Western Australia, Crawley, Australia

**Keywords:** Shear wave elastography, B mode Ultrasound, Idiopathic inflammatory myopathies, Imaging, Myositis

## Abstract

**Background:**

B mode ultrasound (US) and shear wave elastography (SWE) are easily accessible imaging tools for idiopathic inflammatory myopathies (IIM) but require further validation against standard diagnostic procedures such as MRI and muscle biopsy.

**Methods:**

In this prospective cross-sectional study we compared US findings to MRI and muscle biopsy findings in a group of 18 patients (11 F, 7 M) with active IIM (dermatomyositis 6, necrotising autoimmune myopathy 7, inclusion body myositis 4, overlap myositis 1) who had one or both procedures on the same muscle. US domains (echogenicity, fascial thickness, muscle bulk, shear wave speed and power doppler) in the deltoid and vastus lateralis were compared to MRI domains (muscle oedema, fatty infiltration/atrophy) and muscle biopsy findings (lymphocytic inflammation, myonecrosis, atrophy and fibro-fatty infiltration). A composite index score (1–4) was also used as an arbitrary indicator of overall muscle pathology in biopsies.

**Results:**

Increased echogenicity correlated with the presence of fatty infiltration/atrophy on MRI (*p* = 0.047) in the vastus lateralis, and showed a non-significant association with muscle inflammation, myonecrosis, fibrosis and fatty infiltration/atrophy (*p* > 0.333) Severe echogenicity also had a non-significant association with higher composite biopsy index score in the vastus lateralis (*p* = 0.380). SWS and US measures of fascial thickness and muscle bulk showed poor discrimination in differentiating between pathologies on MRI or muscle biopsy. Power Doppler measures of vascularity correlated poorly with the presence of oedema on MRI, or with inflammation or fatty infiltration on biopsy. Overall, US was sensitive in detecting the presence of muscle pathology shown on MRI (67–100%) but showed poorer specificity (13–100%). Increased echogenicity showed good sensitivity when detecting muscle pathology (100%) but lacked specificity in differentiating muscle pathologies (0%). Most study participants rated US as the preferred imaging modality.

**Conclusions:**

Our findings show that US, in particular muscle echogenicity, has a high sensitivity, but low specificity, for detecting muscle pathology in IIM. Traditional visual grading scores are not IIM-specific and require further modification and validation. Future studies should continue to focus on developing a feasible scoring system, which is reliable and allows translation to clinical practice.

**Supplementary Information:**

The online version contains supplementary material available at 10.1186/s41927-022-00276-w.

## Introduction

Idiopathic inflammatory myopathies (IIM) are a heterogeneous group of rare autoimmune diseases that carry a significant morbidity from musculoskeletal complications, as well as potentially serious systemic manifestations in some cases [1]. Early diagnosis is important to allow prompt intervention and improve outcomes.

The gold standard test for the diagnosis of IIM currently remains the muscle biopsy. Varying combinations of muscle inflammation, myofibre necrosis, degeneration, atrophy, and fatty infiltration/fibrosis are typically reported biopsy findings in the IIMs [2, 3] Less invasive methods to assess muscle integrity, such as imaging modalities do have some clinical utility, and magnetic resonance imaging (MRI) is currently regarded as the most sensitive screening modality to identify the presence of muscle pathology in the IIMs [4]. Muscle oedema, atrophy and fatty infiltration are MRI features that are considered to be indicative of muscle inflammation and damage [5].

Clinical led point-of-care ultrasound (US) has rapidly progressed to become a useful imaging outcome tool for various rheumatological conditions [6, 7]. However, there have been relatively few studies of muscle ultrasound in inflammatory muscle diseases, and the role of ultrasound in the diagnosis and monitoring of different varieties of IIM has still not been clearly defined. Moreover, there is a lack of studies that have attempted to compare ultrasound findings with MRI and muscle biopsy changes in different forms of myositis or to evaluate the changes in different ultrasound domains over the course of these diseases, and in response to treatment. Shear wave elastography (SWE), a relatively novel form of US that provides a measure of changes in muscle stiffness, has recently been introduced as a potential complementary tool to traditional B mode US when investigating the limb muscles but requires further investigation in myositis [8, 9]. The validation of US against other diagnostic modalities such as MRI and muscle histopathology in IIM is an essential requirement in facilitating translation of its use into clinical practice.

In this study, we have attempted to correlate changes in muscle ultrasound domains with MRI and muscle biopsy findings in patients with different forms of IIM. The primary objective of the study was to assess the construct and criterion validity of different muscle US domains (viz. echogenicity, muscle bulk, shear wave speed (SWS and power Doppler) against (i) MRI findings of muscle oedema, fatty infiltration/atrophy; and/or (ii) muscle biopsy findings (viz. lymphocytic inflammation, myonecrosis, atrophy, interstitial fibrosis and fatty infiltration) in a mixed group of IIM patients. A secondary objective of the study was to assess intra-observer reliability testing and patient satisfaction with US compared with MRI.

## Methods and methodology

### Participants

Twenty-five patients with a new diagnosis of IIM were recruited sequentially from the rheumatology clinics at Fiona Stanley Hospital and the myositis clinic at the Institute for Immunology and Infectious Diseases at Murdoch University between June 2019 and May 2021. All participants gave written informed consent.

### Recruitment

Human Research Ethics Committee (HREC) approvals were granted by the South Metropolitan Health Service (HREC) (RGS0000003714) and reciprocal ethics were obtained from Murdoch University (2018/237). Patients were required to be ≥ 18 years old, able and willing to provide written informed consent and attend a clinical study visit at a neuromuscular centre. IIM patients were eligible for the study if they satisfied the 2017 European League Against Rheumatism (EULAR) classification of IIM [10] and/or 188th Neuromuscular Centre (ENMC) diagnostic criteria for IBM [11] and/or ENMC 2004 diagnostic criteria for immune-mediated Necrotising Autoimmune Myopathy (NAM) [12]. Patients were recruited within three months of diagnosis to enable comparison with other investigations.

### Clinical assessment

**Fig. 1:**
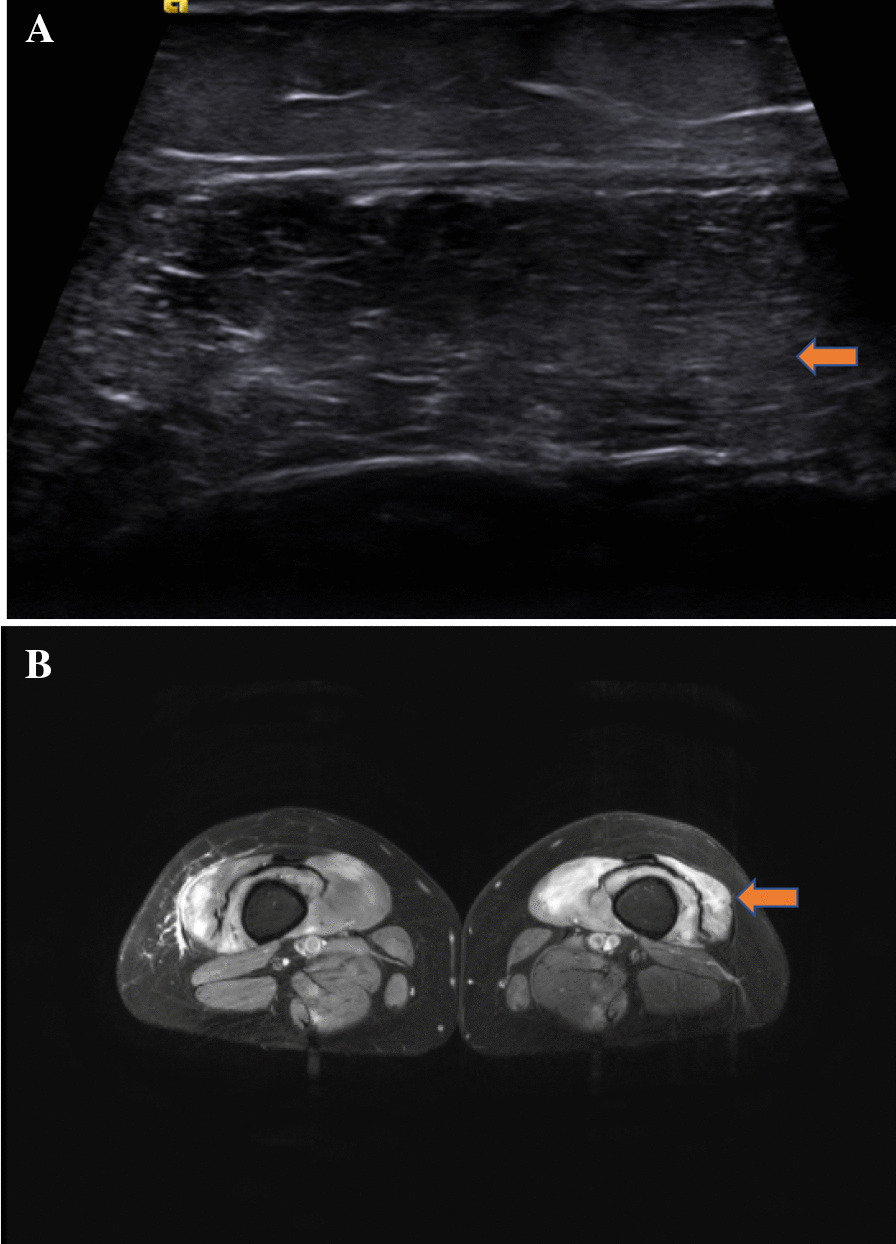
**A** US image showing patchy mild changes in echogenicity with hypoechoic areas and corresponding subcutaneous echogenicity in the vastus lateralis (cross-section) in a 26-year-old with Anti-NXP2. **B** The corresponding MRI in the same patient showed T2 (fat suppression) signal uptake indicative of muscle oedema in the vastus lateralis (arrowheads)

**Fig. 2:**
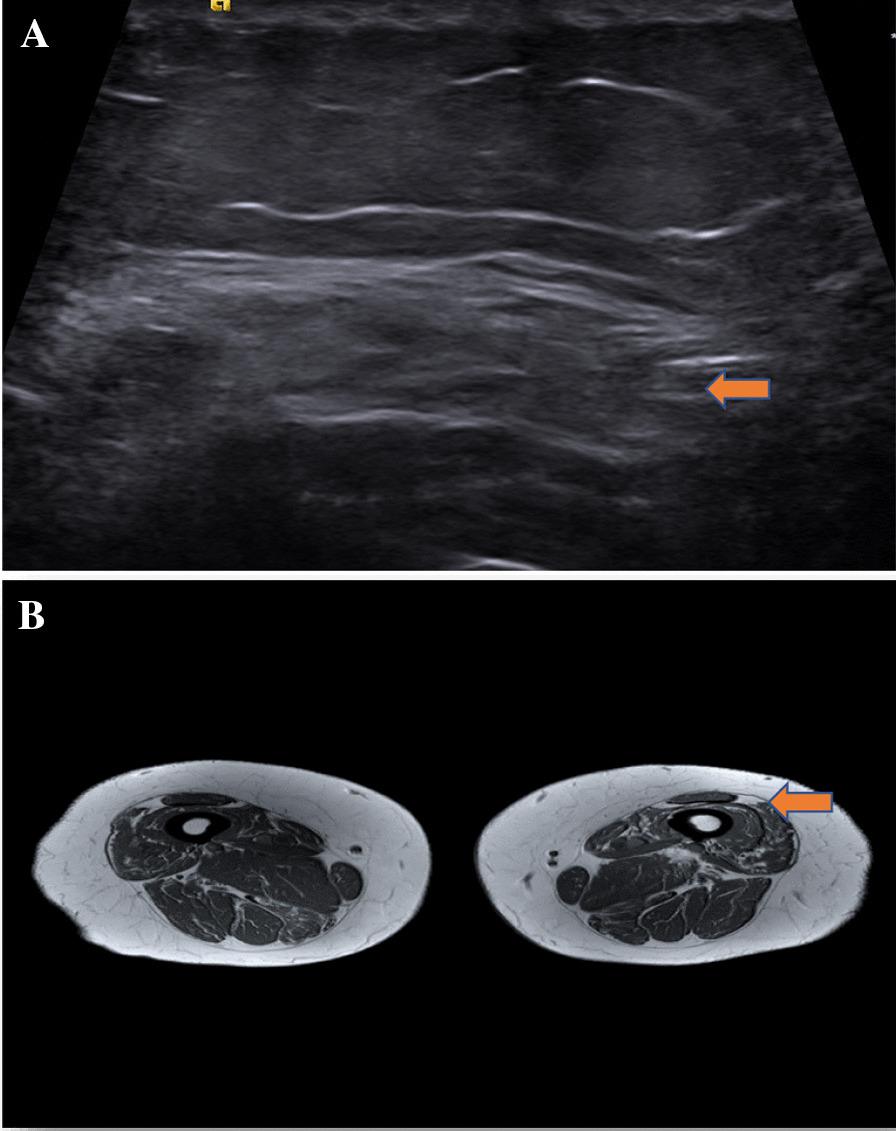
**A** US showing more heterogenous marked echogenicity in the vastus lateralis in a patient with IBM. **B** Corresponding MRI showing T1 (not fat-suppressed) uptake in the vastus lateralis in a patient with IBM

All participants (n = 25) completed patient-reported questionnaires, including a health assessment questionnaire (HAQ), a partially validated tool looking at the functional disability index [13]. A higher index indicates higher functional disability or disease activity. All patients (n = 25) had a clinical examination with manual muscle testing (MMT) in 26 muscle groups [14]. The MMT 26 is a partially validated tool with a total score of 260. The lower the score, the weaker the patient. All patients (n = 25) had a serum creatine kinase (CK) assay as well as other routine biochemical and haematological investigations [15]. As part of their diagnostic workup, 16 patients had MRI of the proximal upper and lower limb muscles, and 13 had a muscle biopsy, which was used as comparators for the US findings (Figs. 1 and 2). The 7 patients without muscle biopsies were from the IBM and anti-HMGCR associated NAM groups, the diagnosis in these cases being based on the clinical phenotype and serological findings. All patients were asked to fill in a patient satisfaction survey for US and MRI.

### Imaging/anatomical landmarks

US and SWE were performed by the same clinician (SP), who had 6 months of supervised training in shear wave elastography (SWE) and 5 years of B mode US experience. In all patients, US and SWE were routinely performed on the left deltoid and right vastus lateralis muscles. The deltoid and vastus lateralis muscles are known to be commonly affected in the IIMs and are the most frequently targeted with MRI and muscle biopsy [16].

### B mode ultrasound technique and protocol

The ultrasound studies were performed in a controlled temperature air-conditioned room. Patients rested for a period of ~ 15 min before the procedure and had been instructed not to perform recreational exercise on the day of the procedure or the preceding day.

B mode US was done using a Siemens machine with 750PRF and 14 MHz. The probe position for imaging the deltoid was at a point one-third of the distance from the acromion to the lateral epicondyle. The arm was flexed in a 90° position held at rest on a pillow. The probe position for imaging the vastus lateralis was at a point one-quarter of the distance from the anterior superior iliac spine (ASIS) to the superior border of the patella [17]. The vastus lateralis was scanned with the knee extended on the bed. Both transverse and longitudinal views of the deltoid and vastus lateralis were obtained. Two images were obtained in each plane and scored.

The B mode features scored were:

1. Fascial thickness was measured in transverse position using the calliper function, with a total of 6 readings in 2 frames with the callipers placed 0.25 cm apart at the most homogenous area of the muscle and averaged [15]. Fascia was defined as the aggregate of connective tissue enveloping the muscle [18] (Additional file 2: Fig. S2).
2. Muscle bulk was measured at the broadest point of the muscle belly, from the deep peripheral fascia to the deep intramuscular fascia using the calliper function [15] (Additional file 2: Fig. S2).
3. Echogenicity, using the Heckmatt visual grading scale (ordinal grade of 1–4) [19], which is a 4 point semi-quantitative scale: 1 = normal, 2 = echogenicity with preservation of bone echo, 3 = echogenicity with partial loss of bone echo and 4 = echogenicity with complete loss of bone echo [19]. A modified version was used where the image was studied in both transverse and longitudinal planes. Higher grade scores correlate with higher echogenicity. For statistical analysis, echogenicity was recorded dichotomously as present/absent, and also graded: normal (grade 1), mild (grade 2) and severe (grade 3–4).

### Power Doppler (PD)

PD was assessed at rest in both the transverse and longitudinal planes. The PD signal was graded as: ‘normal’, ‘mild’, ‘severe’ (Additional file 5: Table S3).

### Shear wave elastography

This was initially performed using the transverse probe position, but during the course of the study the longitudinal plane was also included in view of some studies showing increased reliability with this orientation [20]. SWE was performed using the same Siemens US machine with dual B mode and shear wave capabilities. Minimal contact probe pressure was ensured. Two images with 3 readings in each image were taken using an ROI of 10 mm^2^. The average reading was recorded. This was done for both the deltoid and vastus lateralis.

### MRI

T1 and STIR images on MRI were used to score oedema and fatty infiltration/atrophy. Muscle oedema reported on MRI is presumed to represent inflammation, while fatty infiltration/atrophy are considered to reflect myofibre loss and damage in the muscle [5]. Muscle oedema and fatty infiltration/atrophy were scored dichotomously as present/absent.

### Muscle biopsy

Both light and electron microscopy were performed on the muscle biopsy samples. The major pathological features reported on muscle biopsy comprised: (i) interstitial lymphocytic inflammatory infiltrates; (ii) myofibre necrosis/regeneration; (iii) interstitial fibrosis; and (iv) fatty infiltration/atrophy, collectively being indicators of the extent, severity and chronicity of damage in individual muscles. In addition, to enable comparisons between ultrasound findings and overall muscle pathology, an arbitrary composite pathology index score (1–4) was calculated for each biopsy based on how many of the above four pathology domains were present in individual biopsies.

### Patient satisfaction survey

All participants were asked to fill in a patient satisfaction survey and were required to answer seven questions on their level of satisfaction with US and MRI, respectively. The overall satisfaction with US and MRI were rated separately on a 5-point Likert Scale (1-very good, 2-good, 3-barely acceptable, 4-poor and 5-very poor). An additional question was tabulated regarding preference for imaging modality, where there were 3 options (US, MRI or either) available.

### Intra-observer reliability

The echogenicity and power Doppler US domains in 15 of the participants were re-assessed by the first author for intra-observer reliability at an interval of 8 months after recruitment of the last patient.

### Statistical analysis

Mean (M), standard deviation (SD), median (Mdn) and interquartile ranges (IQR) were used for continuous data. Categorical data were presented using frequency and percent (%). For statistical analysis, echogenicity was considered increased when it showed a grade of 2–4 according to the Heckmatt visual grading scale. PD was considered present when it showed a grade of 1–4. The sensitivity and specificity of US were estimated using MRI and muscle biopsy as the gold-standard comparators. Differences in the categorical variables were explored using Fischer’s exact test (echogenicity and PD) or a Paired-Samples *t*-test (or non-parametric equivalent) (for fascial thickness, muscle bulk and SWS) or reported descriptively. The Mann Whitney U test was used for the comparison between ultrasound and MRI domains. Intra-observer reliability was reported with a kappa value. Patient preference for US was described as the overall percentage.

## Results

### Descriptive demographic data

Following an initial screening, 20 of the 25 patients recruited were considered eligible for inclusion in the study. The 5 patients excluded were initially thought to have a clinical diagnosis of IBM, but after investigation, they did not fulfil the diagnostic criteria for the disease. The median age was 64.0 (IQR: 54.0–70.0) with more females (n = 11, 55.0%) than males (n = 9, 45.0%). Six (30%) participants had a diagnosis of DM, 7 (35%) of NAM, 6 (30%) of IBM, and one of OM (5%). The mean serum CK level at baseline was 4518 U/L (SD = 6036.04). The mean MMT score in the deltoid was 9 (SD = 2.69) and in the vastus lateralis was 7 (SD = 2.77). Eleven participants (55.0%) had both an MRI and muscle biopsy, five (25.0%) had only an MRI, 2 (10.0%) had only a muscle biopsy, and 2 (10.0%) had neither. The median time interval between US and MRI is 53 days (IQR: 15–109) and the median time interval between the US and muscle biopsy is 41 days (21–116). Of the 16 patients who had MRI, 12 had the vastus lateralis imaged, 2 had both the deltoid and vastus lateralis imaged, and 2 had the deltoid only. Of the 13 patients who had a biopsy, 12 had the vastus lateralis biopsied, and 1 had the deltoid biopsied. The two patients who did not have either MRI or muscle biopsy were elderly individuals with IBM who had a typical pattern of muscle weakness and other laboratory findings (serum CK, serology, electromyography) and their data (including US data) were included in the demographic analysis but not the comparative analysis. (Table 1) (Additional file 3: Table S1, Additional file 1: Fig. S1).

**Table 1:** Demographics of IIM participant

Participant	Symptom duration	Time since diagnosis	Age	Sex	Diagnosis	Myositis specific antibodies	CK level	MMT Deltoid/10	MMT Vastus Lateralis/10	Medications at the time of US	Echo deltoid (1–4)	Echo vastus lateralis (1–4)	PD Deltoid (0–4)	PD vastus lateralis (0–4)	MRI deltoid	MRI vastus lateralis	Muscle biopsy deltoid	Muscle biopsy vastus lateralis
1	< 1 yr	< 1 mth	30	F	NAM	Seronegative	9350	9	9	Prednisolone, methotrexate	1	2	1	1	Oedema	N/A	N/A	N/A
2	< 1 mth	< 1 mth	54	F	NAM	Anti-HMGCR	9390	8	9	Prednisolone, methotrexate	2	2	0	0	N/A	Oedema	N/A	N/A
3	< 6 mth	< 1 mth	66	F	NAM	Seronegative	10,600	9	10	Nil	2	3	0	0	N/A	Fatty infiltration	N/A	Atrophy
4	< 1 yr	< 6 mth	79	F	DM	Anti-OJ, Anti-NXP2	553	9	9	Nil	1	2	0	1	N/A	N/A	N/A	Fatty infiltration
5	< 5 yrs	< 1 mth	70	F	IBM	Seronegative	N/A	9	9	Nil	2	3	0	1	N/A	Atrophy, fatty infiltration	N/A	N/A
6	≥ 5 yrs	< 1 mth	60	F	OM	Anti-Ro-52, Anti-NXP2	N/A	10	8	Nil	2	3	2	0	N/A	Oedema	N/A	Inflammation, fibrosis, fatty infiltration
7	< 5 yrs	< 1 mth	74	M	IBM	Seronegative	359	10	N/A	Nil	1	2	0	1	N/A	N/A	N/A	N/A
8	< 1 mth	< 1 mth	20	M	DM	Anti-Mi2a	14,900	1	3	Prednisolone	1	2	3	0	N/A	Oedema	N/A	Inflammation, fibrosis, fatty infiltration
9	≥ 5 yrs	< 1 yr	60	M	DM	Anti-Ro-52, Anti-NXP2	6410	1	1	Prednisolone, mycophenolate	1	1	1	2	N/A	Oedema, fatty infiltration	N/A	N/A
10	< 5 yrs	< 6 mths	70	M	IBM	Seronegative	N/A	10	8	Nil	1	4	0	0	N/A	N/A	N/A	Inflammation, fibrosis, fatty infiltration
11	< 5 yrs	< 1 mth	63	M	IBM	Anti-CN1A	261	10	2	Nil	1	3	2	0	N/A	N/A	N/A	Inflammation, fibrosis, fatty infiltration
12	< 1 yr	< 6 mths	65	M	IBM	Seronegative	1070	10	9	Nil	2	3	0	0	N/A	Oedema, fatty infiltration	N/A	Inflammation, fibrosis, fatty infiltration
13	< 1 yr	< 1 mth	26	F	DM	Anti-NXP2	287	8	9	Prednisolone, methotrexate	1	2	1	0	N/A	Oedema	N/A	Inflammation
14	≥ 5 yrs	< 1 mth	69	M	NAM	Anti-SRP	436	10	8	Nil	1	2	1	2	N/A	N/A	N/A	N/A
15	≥ 5 yrs	< 6 mth	70	M	IBM	Seronegative	69	10	10	Nil	1	1	1	1	N/A	Normal	Normal	N/A
16	< 1 yr	< 1 mth	65	F	NAM	Seronegative	11	10	9	Prednisolone, mycophenolate	3	3	0	0	N/A	Oedema, fatty infiltration	N/A	Inflammation, fatty infiltration
17	< 1 yr	< 1 mth	33	F	NAM	Seronegative	4010	9	7	Prednisolone, hydroxychloroquine, mycophenolate	3	3	0	1	Oedema, atrophy	N/A	Inflammation, fibrosis, fatty infiltration	N/A
18	< 1 yr–< 5 yrs	< 6 mth	54	F	DM	Seronegative	19	10	5	Mycophenolate, prednisolone, IVIG	2	2	0	0	N/A	Oedema	N/A	Fatty infiltration and fibrosis
19	< 6 mth	< 6 mth	61	M	NAM	Anti -HMGCR	19,000	8	5	Prednisolone, methotrexate	1	3	0	0	Oedema	Oedema	N/A	Inflammation, fibrosis, fatty infiltration
20	≥ 5 yrs	< 1 mth	79	F	DM	Anti-SAE	87	9	7	Nil	3	3	1	1	N/A	Atrophy, fatty infiltration	N/A	Inflammation, fibrosis, fatty infiltration
Median/SD	–	–	64/17	–	–	–	553/3	10	8	–	1/1	3/1	0/1	0/1	–	–	–	–

NAM: Necrotising autoimmune myopathy, DM: dermatomyositis, IBM: inclusion body myositis, OM: overlap myositis, CK: creatinine kinase, MMT: manual muscle testing, PD: power Doppler, MRI: magnetic resonance imaging, mths: months, yrs: years, N/A: not available. SD: standard deviation, Echo; echogenicity

### Changes observed with B mode ultrasound

In the deltoid, the median fascial thickness was 0.08 mm (IQR 0.700–0.900), median muscle bulk was 1.54 cm (IQR: 1.19–1.86), median SWS transverse 2.52 m/s (IQR 2.11–2.99), median SWS long m/s 2.70 (IQR: 2.46–2.93). Eleven (11/20, 55%) participants showed increased echogenicity and 11 (11/20, 55%) showed increased vascularity on PD.

In the vastus lateralis, the median fascial thickness was 0.15 (IQR: 0.70–0.90), median muscle bulk was 1.25 cm (IQR: 0.76–1.86), median SWS transverse 2.45 m/s (IQR1.78–3.04) and median SWS long 2.44 m/s (IQR: 1.98–3.22). Eighteen (18/20, 90%) showed increased echogenicity and 11 (11/20, 55%) showed increased vascularity on PD.

### Comparison between US domains and MRI findings

Median values for fascial thickness, muscle bulk and SWS on US were not associated with the presence of oedema, or other changes (e.g., fatty infiltration or atrophy) indicating muscle damage on MRI, in either the deltoid (*p* > 0.400) or vastus lateralis (*p* > 0.370). In the vastus lateralis, there was a non-significant association between increased muscle bulk on US and muscle oedema on MRI (*p* = 0.240), and reduced muscle bulk on US and fatty infiltration/atrophy on MRI (*p* = 0.223) (Additional file 4: Table S2). The grade of echogenicity on US did not reliably differentiate between the presence of inflammation or other features of muscle damage on MRI in the deltoid (*p* > 0.082). However, high-grade echogenicity with associated with fatty infiltration and atrophy in the vastus lateralis (*p*-0.047). There was a poor association between PD signal intensity and the presence of oedema reported on MRI in both the deltoid and vastus lateralis (Additional file 5: Table S3).

### Comparison between US domains and muscle biopsy findings

In the vastus lateralis, the median values for fascial thickness, muscle bulk and SWS did not differentiate between the presence or absence of inflammation, necrosis, fibrosis, or fatty infiltration/atrophy on muscle biopsy (*p* > 0.120) (Additional file 6: Table S4). Although not reaching statistical significance due to the low numbers, increased echogenicity on US was associated with the presence of inflammation (*p* = 1.000), necrosis (*p* = 1.000), fibrosis (*p* = 0.406) and fatty infiltration/atrophy (*p* = 0.333) in biopsies. Similarly, although not reaching statistical significance, it is interesting to note that higher grade echogenicity was associated with more pathologies on biopsy (*p* = 0.380). There was no association between vascularity on PD and the various pathologies on muscle biopsy (*p* > 0.318) (Additional file 7: Table S5).

As the deltoid muscle was biopsied in only one case, we could not adequately compare the continuous and categorical variables in this muscle.

### Sensitivity and specificity of US

Data are presented in Table 2. When using increased echogenicity as the leading US domain in the deltoid, the US had a high sensitivity rate at detecting the presence of muscle pathology on MRI (67–100%) and showed good specificity (75–100%). As only one deltoid was biopsied, the data was omitted. In the vastus lateralis, US was highly sensitive in detecting muscle pathology on MRI (83–90%) and on muscle biopsy (100%) but lacked specificity (13–25%) on MRI and (0%) on muscle biopsy.

**Table 2:** Diagnostic accuracy of ultrasound echogenicity compared to MRI and muscle biopsy in the Deltoid

	MRI muscle oedema (%)	MRI atrophy/fatty infiltration (%)	Bx-inflammation (%)	Bx-necrosis (%)	Bx-fibrosis (%)	Bx-atrophy/fatty infiltration (%)
Deltoid sensitivity	67	100	83	83	100	100
Deltoid specificity	100	75	0	0	0	0
Deltoid PPV	100	50	83	83	67	92
Deltoid NPV	50	100	0	0	0	0
Vastus lateralis sensitivity	90	83	100	100	100	100
Vastus lateralis specificity	25	13	0	0	0	0
Vastus lateralis PPV	75	42	83	83	67	92
Vastus lateralis NPV	50	50	0	0	0	0

Bx: Muscle biopsy, PPV: positive predictive value, NPV: negative predictive value, MRI: magnetic resonance imaging

### Intra-observer reliability

For echogenicity, the intra-observer reliability reported a kappa of 0.589 (moderate agreement) in the deltoid and a kappa of 0.881(substantial agreement) in the vastus lateralis. For PD, the intra-observer reliability reported a kappa of 0.714 (important agreement) in the deltoid and a kappa of 0.746 (significant deal) in the vastus lateralis.

### Participant satisfaction sub-analysis

Eighteen of the 20 participants (90%) responded to the participation satisfaction questionnaire. Seventeen participants (94.4%) overall satisfaction with ultrasound and MRI as very good and one (6%) as good. Eleven participants (11/15, 73.3%) rated the US as the preferred imaging modality, 4 (4/15, 26.7%) liked either (US or MRI), with none (0/15, 0%) choosing MRI.

## Discussion

Before B-mode ultrasound can be accepted as a non-invasive diagnostic procedure of choice for IIMs, changes in individual US domains must be validated against current gold standard diagnostic techniques such as MRI and histopathological findings in the muscle examined. Our study has attempted to do this in a mixed clinic cohort of IIM patients, which included all the primary forms of autoimmune myositis (dermatomyositis, overlap myositis, necrotising autoimmune myositis and inclusion body myositis), except polymyositis, whose existence as a separate entity is controversial and rarely diagnosed [1]. The primary objective was to compare individual ultrasound domains against features of inflammation and muscle damage as shown by MRI and muscle biopsy. This is the first study of this type in which a range of US domains has been compared against specific pathological features on muscle biopsy and a composite score reflecting the overall severity of pathological changes in the biopsy.

The median age of the participants in the study was 64 years, which is reflective of the inclusion of specific IIM subtypes such as IBM, which usually presents over the period of 50 [21]. Participants all had mild to moderate degrees of muscle weakness, and all but two had varying degrees of serum CK elevation, as shown in Table 1. Most participants were entered into the study at the initial diagnostic workup, which included either an MRI, muscle biopsy or both investigations. Still, two elderly patients with typical clinical features (history, examination, EMG and CK levels) of sporadic IBM did not have an MRI or muscle biopsy. Myositis-specific autoantibodies were present in 10/20 (50%) participants and helped classify the myositis subtype in these cases [22]. As the classification of IIM continues to evolve, non-invasive investigations such as serological markers are now assuming a more important role in diagnosis. They may avoid the need for muscle biopsy in some cases [22].

Although our findings indicated a correlation between increased echogenicity on the US and fatty infiltration and atrophy on MRI, the degree of echogenicity did not reliably differentiate between inflammation and other features of muscle damage, which brings into question the US grading scale used. The Heckmatt grading scale [19], or modified versions of it, are predominantly used in neuromuscular ultrasound studies, despite being quite dated [23], and do not consider hypoechogenicity as a feature of inflammation. Since then, the Siena group have proposed a new greyscale and power doppler scoring system for IIMs [24], which predominantly uses hypoechogenicity as an indicator of oedema and hyperechogenicity as an indicator of muscle damage. However, as this was a construct validity study, it was not designed to interrogate the criterion validity of this newly proposed grading scale. This grading scale was not included in the present study. Our study commenced before the new proposed Siena grading criteria were released. Different reference muscles were used in the two studies (e.g. rectus femoris in the Siena study and vastus lateralis in the present study). It is also unclear whether the vastus intermedius, as proposed by the Siena group, can be used as a normal healthy comparator to a diseased muscle, as the vastus intermedius may also be involved in the IIMs. Although not reaching statistical significance, our finding of increased muscle bulk on the US in muscles showing oedema on MRI, and conversely of reduced muscle bulk when MRI showed changes of chronic muscle damage (i.e. fibrosis and fatty infiltration), are intuitively concordant and also align with previous literature [18].

Because of our small sample sizes and heterogeneity of biopsy findings in participants with different forms of IIM, the comparison of US findings with histopathological features placed more emphasis on descriptive observations. Nevertheless, a non-significant association between high echogenicity and histological features of inflammation, myonecrosis and fatty infiltration/atrophy was observed in the vastus lateralis. This is interesting, as previous reports have suggested that hypoechogenicity is associated with the presence of oedema on MRI, particularly in early, more active forms of myositis [19]. Our findings indicate that from a pathological standpoint, these changes are all combined to varying degrees in individual patients depending on the disease duration, IIM subtype and activity of the disease at the time of the investigations. A possible explanation for higher echogenicity in our study is the inclusion of patients with more chronic forms of IIM, such as sporadic IBM. Apart from increased echogenicity, other US domains such as fascial thickness, muscle bulk, and SWS did not correlate with muscle histological changes.

Increased power Doppler signal, indicative of increased vascularity, has previously been associated with muscle oedema and early, more active myositis [17, 19]. However, in the more chronic IIM cohort, we found a poor correlation between PD signal and oedema on MRI or inflammation and fatty infiltration on muscle biopsy. Increased vascularity is assumed to occur in the presence of acute inflammation, correlating with disease activity [24, 25]. The disparity between our PD findings and some previous studies is likely to be due to differences in the types of IIM cases included in the study and disease activity and treatment effects at the time of the investigation. Our findings with PD were similar to those in our previous preliminary study, which suggested that the PD score was of limited value in differentiating between pathological and normal muscle and drew attention to the possible confounding effects of exercise, temperature and hyperdynamic states on the PD signal [9].

## Limitations

There are several limitations to the present study. Firstly, the small sample size and heterogeneity of the IIM cohort are potential limitations that could affect the significance and generalisability of the findings. Due to the low prevalence of IIMs, future studies will need to consider multi-site data collection or extend the data collection period to overcome this issue. Secondly, ultrasound remains operator-dependant, and the lack of standardised protocols and consensus agreements on what constitutes features of inflammation and muscle damage may influence the reliability of the findings. Thirdly, the MRI and muscle biopsies were not all prospective and coincident in time. Biopsies were not MRI-guided, which may have reduced the strength of some of the associations found between the imaging and pathology domains. The MRI was scored dichotomously in this study, but other ways to achieve pathology on MRI also warrant further investigation.

## Conclusions

Ultrasound has shown high sensitivity in detecting muscle pathology in IIM, but the discrimination of pathology domains and the utility of different US domains are still contentious. To further improve specificity and reliability, definitions of muscle inflammation and damage on US would need to be refined and scoring systems matched against gold standard techniques to interrogate the validity of the findings in a larger IIM patient cohort having all three procedures. Despite this, our study suggests that ultrasound is a valuable and feasible screening imaging tool in IIM, which patients favourably receive, and may also help identify a suitable muscle for biopsy. Moreover, muscle ultrasound may also have utility in other situations, such as detecting muscle involvement in clinically amyopathic dermatomyositis, detecting active myositis in patients with the treated disease, and differentiating steroid myopathy from persisting active myositis.

## Supplementary Information

**Additional file 1**.** Supplementary Figure 1**. Study design.

**Additional file 2**.** Supplementary Figure 2**. Fascial thickness and muscle thickness measurements in a deltoid muscle.

**Additional file 3**.** Supplementary Table 1**. Demographic of the continuous variables.

**Additional file 4**.** Supplementary Table 2**. Ultrasound domains (continuous data) against MRI domains in the vastus lateralis and deltoid.

**Additional file 5**.** Supplementary Table 3**. Ultrasound domains (categorical data) against MRI domains in the deltoid and vastus lateralis.

**Additional file 6**.** Supplementary Table 4**. Ultrasound domains (continuous data) against muscle biopsy domains in the Vastus Lateralis.

**Additional file 7**.** Supplementary Table 5**. Ultrasound domains (categorical data) against muscle biopsy in the Vastus Lateralis.

## Data Availability

All data and materials are available in this publication in the Additional files 3–7.

## Abbreviations

IBM: Inclusion body myositis
NAM: Necrotising autoimmune myositis
MMT: Manual muscle testing
PD: Power Doppler
EMG: Electromyography
US: Ultrasound
MRI: Magnetic resonance imaging
SWE: Shear wave elastography
SWS: Shear wave speed
IIM: Idiopathic inflammatory myopathies
HAQ: Health assessment questionnaire
VAS: Visual analogue scale
CK: Creatine kinase
