# Correction to: Muscle B mode ultrasound and shear-wave elastography in idiopathic inflammatory myopathies (SWIM): criterion validation against MRI and muscle biopsy findings in an incident patient cohort

**DOI:** 10.1186/s41927-022-00302-x

**Published:** 2022-08-30

**Authors:** Shereen Paramalingam, Merrilee Needham, Sarah Harris, Susan O’Hanlon, Frank Mastaglia, Helen Keen

**Affiliations:** 1 University of Notre Dame Australia, Fremantle, WA Australia; 2 Department of Rheumatology, Fiona Stanley Hospital, 11 Robin Warren Dr, Murdoch, WA 6150 Australia; 3 Institute for Immunology and Infectious Diseases, Murdoch University, Murdoch, WA Australia; 4 Department of Neurology, Fiona Stanley Hospital, Murdoch, WA Australia; 5 Institute for Health Research, Notre Dame Australia, Fremantle, WA Australia; 6 Western Australian Health Translation Network (WAHTN), Bentley, WA Australia; 7 Department of Radiology, Fiona Stanley Hospital, Murdoch, WA Australia; 8 Present Address: Perron Institute for Neurological and Translational Science, University of Western Australia, Crawley, Australia

## Correction to: BMC Rheumatology (2022) 6:47. 10.1186/s41927-022-00276-w

Following publication of the original article [1], it was reported that there was an error in the caption for **Fig. 1A**.

The caption previously read: “**Fig. 1A** US image showing patchy mild changes in echogenicity with hypoechoic areas and corresponding subcutaneous echogenicity in the vastus lateralis (cross-section) in a 26-year-old with Anti-NXP2.”

The correct caption is: “**Fig. 1A** Mild increased echogenicity in the vastus lateralis (cross section) and subcutaneous tissue with patches of hypoechogenicity in a 26-year-old with Anti-NXP2 DM.”

The article also contained several typographical errors, which have been corrected.

The original article [1] has been updated.
