# Supplementary material for: Muscle B mode ultrasound and shear-wave elastography in idiopathic inflammatory myopathies (SWIM): criterion validation against MRI and muscle biopsy findings in an incident patient cohort
Source: BMC Rheumatol. 2022 Aug 8;6:47. doi: 10.1186/s41927-022-00276-w (PMC9358818; doi:10.1186/s41927-022-00276-w)
Supplement: Supplementary file 1 — Additional file 1. Supplementary Figure 1. Study design. [file 41927_2022_276_MOESM1_ESM.docx]

**Supplementary Figure 1: Study design**

Diagnostic Test

Clinical Examination

HAQ: Health assessment questionnaire, VAS: Visual analogue scale, MMT: manual muscle testing, CK: creatine kinase, MRI: magnetic resonance imaging, US: ultrasound, SWE: shear wave elastography
