# Supplementary figures and images for: Muscle B mode ultrasound and shear-wave elastography in idiopathic inflammatory myopathies (SWIM): criterion validation against MRI and muscle biopsy findings in an incident patient cohort
Source: BMC Rheumatol. 2022 Aug 8;6:47. doi: 10.1186/s41927-022-00276-w (PMC9358818; doi:10.1186/s41927-022-00276-w)

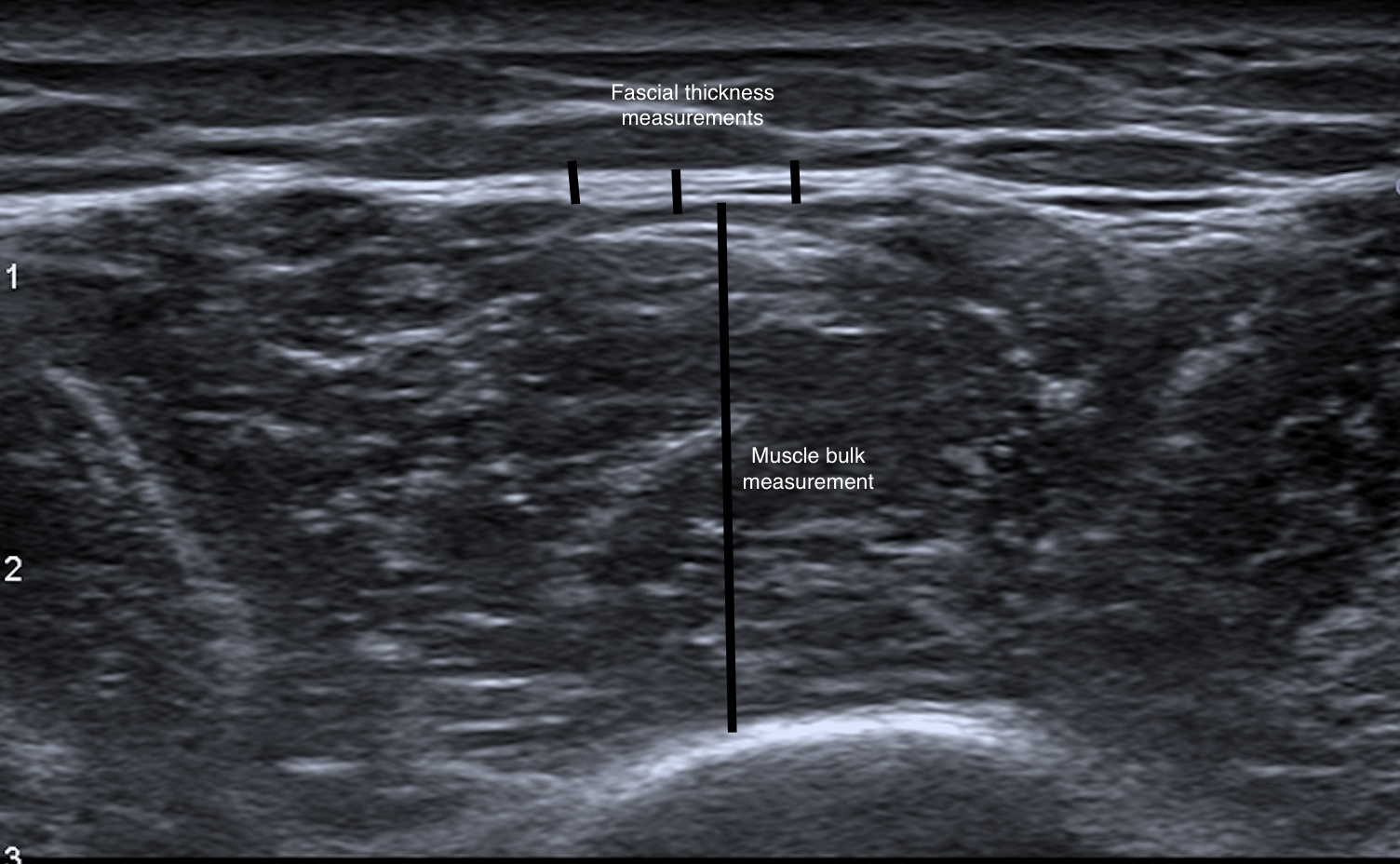

Supplement: Supplementary file 2 — Additional file 2. Supplementary Figure 2. Fascial thickness and muscle thickness measurements in a deltoid muscle. [file 41927_2022_276_MOESM2_ESM.png]
