# Supplementary material for: Muscle B mode ultrasound and shear-wave elastography in idiopathic inflammatory myopathies (SWIM): criterion validation against MRI and muscle biopsy findings in an incident patient cohort
Source: BMC Rheumatol. 2022 Aug 8;6:47. doi: 10.1186/s41927-022-00276-w (PMC9358818; doi:10.1186/s41927-022-00276-w)
Supplement: Supplementary file 3 — Additional file 3. Supplementary Table 1. Demographic of the continuous variables. [file 41927_2022_276_MOESM3_ESM.docx]

**Supplementary Table 1:** Demographic of the continuous variables

|  | Mean | Median | Std Dv | IQR |
| --- | --- | --- | --- | --- |
| HAQ | 0.89 | 0.75 | 0.77 | 0.20-1.38 |
| CK | 4518.88 | 553.00 | 6036.04 | 174.00-9370.00 |
| MMT/10  Deltoid | 8.55 | 9.50 | 2.69 | 8.25-10.00 |
| MMT/10  Vastus  lateralis | 7.26 | 8.00 | 2.77 | 5.00-9.00 |
| FT  Deltoid  Average/mm | 0.08 | 0.08 | 0.25 | 0.700-0.900 |
| FT  Vastus lateralis average/mm | 0.12 | 0.15 | 0.03 | 0.09-0.138 |
| Muscle bulk  Deltoid/cm | 1.52 | 1.54 | 0.43 | 1.19-1.86 |
| Muscle bulk  Vastus lateralis/cm | 1.31 | 1.25 | 0.63 | 0.76-1.84 |
| SWS  Deltoid  Rest/m/s | 2.45 | 2.52 | 0.70 | 2.11-2.99 |
| SWS  Deltoid rest long/m/s | 2.74 | 2.70 | 0.33 | 2.46-2.93 |
| SWS  Vastus  Lateralis  Rest/m/s | 2.68 | 2.45 | 1.43 | 1.78-3.04 |
| SWS  Vastus  Lateralis long /m/s | 2.40 | 2.44 | 0.72 | 1.98-3.22 |

Std Dv: standard deviation, IQR: interquartile range, HAQ: health assessment questionnaire, ck: creatinine kinase, MMT: manual muscle testing, FT: fascial thickness, D: deltoid, VL: vastus lateralis, SWS: shear wave speed
