# Supplementary material for: Muscle B mode ultrasound and shear-wave elastography in idiopathic inflammatory myopathies (SWIM): criterion validation against MRI and muscle biopsy findings in an incident patient cohort
Source: BMC Rheumatol. 2022 Aug 8;6:47. doi: 10.1186/s41927-022-00276-w (PMC9358818; doi:10.1186/s41927-022-00276-w)
Supplement: Supplementary file 4 — Additional file 4. Supplementary Table 2. Ultrasound domains (continuous data) against MRI domains in the vastus lateralis and deltoid. [file 41927_2022_276_MOESM4_ESM.docx]

**Supplementary Table 2.** Ultrasound domains (continuous data) against MRI domains in the vastus lateralis and deltoid

| Ultrasound domains | Inflammation | | | Damage | | |
| --- | --- | --- | --- | --- | --- | --- |
|  | Oedema  Present  (mdn/Std) | Oedema  Absent  (Mdn/Std) | Mann-Whitney U/Standardised  Test statistic/Exact sig | Fatty infiltration  or atrophy  Present  (Mdn/Std) | Fatty  Infiltration  or atrophy  Absent  (Mdn/Std) | Mann-Whitney  U/Standardised Test statistic/Exact sig |
| VL Fascial thickness/mm | 0.13/0.03 | 0.16/0.40 | 23.00/0.431/0.73 | 0.13/0.12 | 0.13/0.41 | 20.00/-0.53/0.66 |
| VL Muscle bulk/cm | 1.64/0.54 | 1.28/0.37 | 11.00/-1.27/0.24 | 1.05/0.25 | 1.78/0.42 | 34.00/1.29/0.23 |
| VL SWS rest transverse (m/s) | 2.18/1.70 | 3.04/2.31 | 18.00/1.28/0.26 | 2.13/2.00 | 2.85/2.10 | 12.00/0.00/1.00 |
| VL SWS rest long (m/s) | 2.03/0.86 | 2.44/0.93 | 17.00/1.01/0.35 | 1.36/1.16 | 2.25/0.56 | 14.00/0.426/0.76 |
| D Fascial thickness/mm | 0.09/0.00 | * | 0.00/-1.41/0.50 | * | 0.75/0.02 | 0.50/-1.01/0.40 |
| D Muscle bulk/cm | 1.68/0.60 | * | 1.00/-0.45/1.00 | * | 1.74/0.51 | 2.00/0.00/1.00 |
| D SWS rest transverse (m/s) | 2.56/1.19 | * | 3.00/1.34/0.50 | * | 1.94/1.25 | 2.00/0.00/1.00 |
| D SWS rest long (m/s) |  | * | 3.00/1.34/0.50 | * |  | 1.00/-0.71/0.80 |

VL: vastus lateralis, D: deltoid, MRI: magnetic resonance imaging, SWS: shear wave speed, Mdn: median, Std: standard deviation

* Unable to calculate as n=1
