# Supplementary material for: Muscle B mode ultrasound and shear-wave elastography in idiopathic inflammatory myopathies (SWIM): criterion validation against MRI and muscle biopsy findings in an incident patient cohort
Source: BMC Rheumatol. 2022 Aug 8;6:47. doi: 10.1186/s41927-022-00276-w (PMC9358818; doi:10.1186/s41927-022-00276-w)
Supplement: Supplementary file 5 — Additional file 5. Supplementary Table 3. Ultrasound domains (categorical data) against MRI domains in the deltoid and vastus lateralis. [file 41927_2022_276_MOESM5_ESM.docx]

**Supplementary Table 3:** Ultrasound domains (categorical data) against MRI domains in the deltoid and vastus lateralis

| US domains |  | Oedema  Present  (n) | Oedema  Absent  (n) | P value  (Fisher Exact 2-sided test) | Fatty infiltration  or atrophy  (n) | Fatty  Infiltration  or atrophy  (n) | P value  (Fisher Exact test 2- sided test) |
| --- | --- | --- | --- | --- | --- | --- | --- |
| D Echogenicity | Normal | 1 | 1 | 0.513 | 0 | 3 | 0.082 |
|  | Mild | 1 | 0 |  | 0 | 1 |  |
|  | Severe | 1 | 0 |  | 1 | 0 |  |
| D Power Doppler- vascularity | Normal | 2 | 0 | 0.500 | 1 | 2 | 1.000 |
|  | Mild | 1 | 1 |  | 0 | 2 |  |
|  | severe | 0 | 0 |  | 0 | 0 |  |
| VL Echogenicity | Anechoic | 1 | 1 | 0.730 | 1 | 1 | **0.047** |
|  | Mild | 4 | 1 |  | 0 | 5 |  |
|  | Severe | 5 | 2 |  | 5 | 2 |  |
| VL Power Doppler- vascularity | Normal | 9 | 0 | **0.005** | 3 | 6 | 0.580 |
|  | Mild | 1 | 4 |  | 3 | 2 |  |
|  | Severe | 0 | 0 |  | 0 | 0 |  |

D: deltoid, VL: vastus lateralis, MRI: magnetic resonance imaging
