# Supplementary material for: Muscle B mode ultrasound and shear-wave elastography in idiopathic inflammatory myopathies (SWIM): criterion validation against MRI and muscle biopsy findings in an incident patient cohort
Source: BMC Rheumatol. 2022 Aug 8;6:47. doi: 10.1186/s41927-022-00276-w (PMC9358818; doi:10.1186/s41927-022-00276-w)
Supplement: Supplementary file 6 — Additional file 6. Supplementary Table 4. Ultrasound domains (continuous data) against muscle biopsy domains in the Vastus Lateralis. [file 41927_2022_276_MOESM6_ESM.docx]

**Supplementary Table 4:** Ultrasound domains (continuous data) against muscle biopsy domains in the Vastus Lateralis

| US domains | Inflammation | | | Damage | | | | | | | | |
| --- | --- | --- | --- | --- | --- | --- | --- | --- | --- | --- | --- | --- |
|  |  |  |  | Necrosis  Present  (Mdn/Std) | Necrosis  Absent  (Mdn/Std) | Mann-Whitney U/Standardised test statistic/Exact sig | Fibrosis  Present  (Mdn/Std) | Fibrosis  Absent  (Mdn/Std) | Mann-Whitney U/Standardised test statistic/Exact sig | Fatty infiltration/  Atrophy  (Mdn/Std) | Fatty infiltration/  Atrophy  (Mdn/Std) | Mann-Whitney U/Standardised test statistic/Exact sig |
|  | Present  (Mdn/Std) | Absent  (Mdn/Std) | Mann-Whitney U/Standardised test statistic/Exact sig |  |  |  |  |  |  |  |  |  |
| Fascial thickness/mm | 0.13/0.43 | * | 7.50/-0.54/0.61 | 0.13/0.43 | * | 7.5/00.54/0.61 | 0.13/0.05 | 0.12/0.02 | 11.5/-0.77/0.46 | 0.13/0.05 | * | 4.00/-0.44/0.83 |
| Muscle bulk/cm | 1.05/0.67 | * | 2.00/-1.72/0.12 | 1.05/0.67 | * | 2.00/-1.72/0.12 | 0.86/0.57 | 1.81,0.64 | 21.0/0.85/0.46 | 0.96/0.53 | * | 10.00/1.30/0.33 |
| SWS rest transverse | 2.39/1.23 | * | 6.00/0.78/0.67 | 2.39/1.23 | * | 6.00/0.78/0.67 | 2.39/1.33 | 1.97/0.96 | 7.0/-0.52/0.71 | 2.26/1.35 | * | 3.00/-0.39/0.89 |
| SWS rest long | 2.07/0.72 | * | 4.00/0.00/1.00 | 2.07/0.72 | * | 4.00/0.00/1.00 | 2.41/0.72 | 1.64/0.60 | 8.0/-0.26/0.91 | 2.20/0.79 | * | 6.00/0.78/0.67 |

Mdn: median, U: Mann Whitney U test, Z: standard error

* Data not available as all vastus lateralis muscle biopsied were abnormal

** Deltoid muscle not displayed as n=1
