# Supplementary material for: Muscle B mode ultrasound and shear-wave elastography in idiopathic inflammatory myopathies (SWIM): criterion validation against MRI and muscle biopsy findings in an incident patient cohort
Source: BMC Rheumatol. 2022 Aug 8;6:47. doi: 10.1186/s41927-022-00276-w (PMC9358818; doi:10.1186/s41927-022-00276-w)
Supplement: Supplementary file 7 — Additional file 7. Supplementary Table 5. Ultrasound domains (categorical data) against muscle biopsy in the Vastus Lateralis. [file 41927_2022_276_MOESM7_ESM.docx]

**Supplementary Table 5:** Ultrasound domains (categorical data) against muscle biopsy in the

Vastus Lateralis

| US domains | Scoring | Inflammation  Present  (n) | Inflammation  Absent  (n) | P value  (Exact-sig) | Necrosis  Present  (n) | Necrosis  Absent  (n) | P value  (Exact-sig) | Fibrosis  Present  (n) | Fibrosis  Absent  (n) | P value  (Exact-sig) | Fat and atrophy  Present  (n) | Fat and atrophy  Absent  (n) | P value (Chi-Square 2-sided) | Grading | CI-1  (n) | CI-2  (n) | CI-3  (n) | CI-4  (n) | P value Chi-Square 2- sided) |
| --- | --- | --- | --- | --- | --- | --- | --- | --- | --- | --- | --- | --- | --- | --- | --- | --- | --- | --- | --- |
| Echogenicity | Normal | 0 | 0 | 1.000 | 0 | 0 | 1.000 | 0 | 0 | 0.406 | 0 | 0 | 0.333 | Normal | 0 | 0 | 0 | 0 | 0.380 |
|  | Mild | 3 | 1 |  | 3 | 1 |  | 2 | 2 |  | 3 | 1 |  | Mild | 1 | 1 | 0 | 2 |  |
|  | Severe | 7 | 1 |  | 7 | 1 |  | 6 | 2 |  | 8 | 0 |  | Severe | 0 | 1 | 2 | 4 |  |
| Power Doppler- vascularity | Normal | 9 | 1 | 0.318 | 9 | 1 | 0.318 | 7 | 3 | 1.000 | 9 | 1 | 1.000 | Normal | 0 | 2 | 2 | 5 | 0.145 |
|  | Mild | 1 | 1 |  | 1 | 1 |  | 1 | 1 |  | 2 | 0 |  | Mild | 1 | 0 | 0 | 1 |  |
|  | Severe | 0 | 0 |  | 0 | 0 |  | 0 | 0 |  | 0 | 0 |  | Severe | 0 | 0 | 0 | 0 |  |

US: ultrasound, CI: composite index

** Deltoid data could not be calculated as n=1
